# Shelby Medical College

**Published:** 1858-07

**Authors:** 


Shelby Medical College. — We perceive from a circular just received, that a new Medical School will be opened next autumn, at Nashville, Tennessee. The session will commence on the 1st of November, and terminate on the 1st of March ensuing. The Faculty, whose names, however, are not yet announced, is to be composed of seven professors, the branches to be taught being such as are usually included in the courses of lectures delivered in this country. The circular is signed by Dr. John P. Ford, one of the most distinguished physicians of Tennessee, as Dean. The report is, that his associates are to be Dr. Haskins, of Clarksville, Dr. Atchison and Dr. Calendar, of Nashville, and Dr. May, of Washington City, with two other gentlemen whose names have not transpired. With such a Faculty, Shelby College cannot fail to obtain speedy distinction. Dr. Haskins is one of the most able chemists in the South-west, and is well known by his valuable contributions to medical science. Dr. Atchison has long enjoyed a high reputation as a practitioner, and Dr. Calendar, although a recent graduate, is a young gentleman of great professional promise. The Trustees have been fortunate in securing Dr. May, whose character as an accomplished surgeon, a skilful operator, and an eloquent teacher is well known throughout the country.

